# Phenotypic comparison and the potential antitumor function of immortalized bone marrow-derived macrophages (iBMDMs)

**DOI:** 10.3389/fimmu.2024.1379853

**Published:** 2024-04-08

**Authors:** Dong-kun Xie, Jin Yao, Peng-hui Li, Yan-wen Zhu, Jia-nuo Chen, Xiu-li Cao, Shi-lin Cheng, Ya-miao Chen, Yi-fei Huang, Liang Wang, Zan-han Wang, Rong Qiao, Jia-mei Ge, Huan Yue, Li Wei, Zhong-yuan Liu, Hua Han, Hong-yan Qin, Jun-long Zhao

**Affiliations:** ^1^ College of Life Sciences, Northwest University, Xi’an, Shaanxi, China; ^2^ State Key Laboratory of Holistic Integrative Management of Gastrointestinal Cancers, Medical Genetics and Development Biology, Fourth Military Medical University, Xi’an, China; ^3^ Department of Orthopedics, Xijing Hospital, Fourth Military Medical University, Xi’an, China; ^4^ Department of Biochemistry and Molecular Biology, Fourth Military Medical University, Xi’an, China

**Keywords:** macrophage, iBMDM, polarization, immunotherapy, antitumor

## Abstract

**Introduction:**

Macrophages are an important component of innate immunity and involved in the immune regulation of multiple diseases. The functional diversity and plasticity make macrophages to exhibit different polarization phenotypes after different stimuli. During tumor progression, the M2-like polarized tumor-associated macrophages (TAMs) promote tumor progression by assisting immune escape, facilitating tumor cell metastasis, and switching tumor angiogenesis. Our previous studies demonstrated that functional remodeling of TAMs through engineered-modifying or gene-editing provides the potential immunotherapy for tumor. However, lack of proliferation capacity and maintained immune memory of infused macrophages restricts the application of macrophage-based therapeutic strategies in the repressive tumor immune microenvironment (TIME). Although J2 retrovirus infection enabled immortalization of bone marrow-derived macrophages (iBMDMs) and facilitated the mechanisms exploration and application, little is known about the phenotypic and functional differences among multi kinds of macrophages.

**Methods:**

HE staining was used to detect the biosafety of iBMDMs, and real-time quantitative PCR, immunofluorescence staining, and ELISA were used to detect the polarization response and expression of chemokines in iBMDMs. Flow cytometry, scratch assay, real-time quantitative PCR, and crystal violet staining were used to analyze its phagocytic function, as well as its impact on tumor cell migration, proliferation, and apoptosis. Not only that, the inhibitory effect of iBMDMs on tumor growth was detected through subcutaneous tumor loading, while the tumor tissue was paraffin sectioned and flow cytometry was used to detect its impact on the tumor microenvironment.

**Results:**

In this study, we demonstrated iBMDMs exhibited the features of rapid proliferation and long-term survival. We also compared iBMDMs with RAW264.7 cell line and mouse primary BMDMs with *in vitro* and *in vivo* experiments, indicating that the iBMDMs could undergo the same polarization response as normal macrophages with no obvious cellular morphology changes after polarization. What’s more, iBMDMs owned stronger phagocytosis and pro-apoptosis functions on tumor cells. In addition, M1-polarized iBMDMs could maintain the anti-tumor phenotypes and domesticated the recruited macrophages of receptor mice, which further improved the TIME and repressed tumor growth.

**Discussion:**

iBMDMs can serve as a good object for the function and mechanism study of macrophages and the optional source of macrophage immunotherapy.

## Introduction

Macrophages are an important component of intrinsic immunity and possess a variety of functions, including homeostasis maintenance, removal of cellular debris, elimination of pathogens and modulation of inflammatory responses (1, 2). In the tumor microenvironment, tumor-associated macrophages (TAMs) participate in immune regulation and tumor angiogenesis to affect tumor development (3–5). Due to the different stimuli in the microenvironment, TAMs present “two-sided” roles with various polarized phenotypes. Macrophages can be activated by interferon gamma (IFNγ) and Toll-like receptor (TLR) agonists to develop an inflammatory (M1-like) phenotype, thus exhibiting proinflammatory characteristics with microbial killing and tumor growth inhibition (6, 7). Conversely, in response to interleukin-4 (IL-4), IL-13 and IL-10 (M2-like activation), macrophages release anti-inflammatory factors, which promote immunosuppression, debris removal, angiogenesis, tissue remodeling and repair (7–9). Investigating the complex cellular mechanisms of macrophages in the context of disease is emerging as a fundamental step in understanding pathogenesis as well as performing macrophage immunotherapy (10).

Considering the pivotal influence of macrophage development and function on disease progression, immunotherapy based on macrophages has achieved some progress in recent years (11–15). Our previous studies have demonstrated that stimulated M1 macrophages and miR-125a-overexpressing macrophages could alleviate liver fibrosis and repress tumor growth, respectively (16). The strong plasticity and functional diversity endow macrophages with better immunotherapeutic effects and advantages. However, macrophage-based therapeutic strategies still face two limitations in terms of antitumor immunity. On the one hand, although engineered modified or gene-edited macrophages exhibit obvious antitumor potential, the repressive tumor immune microenvironment (TIME) accelerates their functional remodeling to limit immunotherapy. On the other hand, the lack of proliferation ability of infused macrophages makes gene editing and cell harvesting more difficult, which increases the treatment time and immunotherapy cost (17). How to solve the problem of microenvironment domestication and lasting proliferation ability has become the focus of macrophage immunotherapy.

Currently, the majority of macrophage sources for basic research and immunotherapy exploration consist of bone marrow-derived macrophages (BMDMs) (17–19), induced pluripotent stem cell-derived macrophages (iPS-Mφ) (20–22) and the RAW264.7 cell line (23–25). BMDMs are fully developed and function regulable and are more suitable for *in vitro* experiments and *in vivo* verification. However, the BMDMs could not achieve stable genotypic transformation due to proliferation limitations. Flexible gene editing and functional modification are advantages of iPS-Mφs in cellular immunotherapy. It is extremely costly and difficult to obtain and culture iPS-Mφs. Meanwhile, it has been reported that iPS-Mφs present an M2-like polarization phenotype, which is not appropriate for tumor immunotherapy (26). RAW264.7 is a kind of fusion-immortalized monocyte-macrophage line of BALB/c mouse origin that was established from murine tumors induced with Abelson leukemia virus by Raschke et al. in 1978 (27). The RAW264.7 cells were only used for some *in vitro* experiments of macrophage function analysis (28). Therefore, it is crucial to seek effective and safe cell sources for macrophage immunotherapy.

A growing amount of evidence highlights the intriguing possibility that macrophage immortalization may be a viable strategy for macrophage-based immunotherapy. J2 retrovirus infection-enabled immortalization has been successfully applied to fetal liver macrophages, spleen macrophages, microglia, and bone marrow-derived macrophages (BMDMs) (29–32). Immortalized macrophages express surface biomarkers of macrophages and possess typical functional characteristics. In addition, they share strong proliferation ability and long-term survival potential. Therefore, gene-edited immortalized macrophages are easy to construct, which facilitates the advancement of macrophage regulatory mechanisms. The study by Iolanda Spera et al. in 2021 detected and analyzed the functions of the immortalized BMDM (iBMDM) cell lineage from a metabolic point of view (33). By determining intracellular and extracellular metabolites as well as the phenotypic characteristics of immortalized versus primary BMDMs, it was concluded that immortalized BMDMs exhibited similar metabolism and polarization characteristics under both classical and alternative stimulation. However, no study has systematically evaluated and compared the biosafety, immunological characteristics and antitumor functions of iBMDMs. In this study, we detected the proliferation efficiency and survival time of iBMDMs both *in vitro* and *in vivo*, indicating that iBMDMs have good biosafety and low immunogenicity. Immunology tests and coculture experiments with tumor cells were used to analyze the effect of iBMDMs on the malignant biological behaviors of tumor cells. Finally, the infusions of different macrophages into tumor-bearing mice suggested that iBMDMs present even stronger antitumor potential than primary BMDMs. Our study comprehensively explores the antitumor functions of iBMDMs *in vitro* and *in vivo* and demonstrates that iBMDMs are an optional source of macrophage immunotherapy.

## Materials and methods

### Animals and tumor models

Wild-type C57BL/6 mice used in this study were maintained in a specific pathogen-free facility. All the animal experiments were approved by the Animal Experiment Administration Committee of the Fourth Military Medical University to ensure the ethical and humane treatment of the animals. And all experiments used 8-week-old to 12-week-old male mice. The LLC cell line was purchased from the authenticated ATCC repository in 2014. LLC was mixed with macrophages at a ratio of 5:1 (5×10^6^:1×10^6^) and injected into the subcutaneous tissue of the backs of mice. The length and width of the tumor tissue were measured using a ruler and analyzed after 3 weeks of coculture. The mice were sacrificed at 2 or 3 weeks after inoculation, and tumors were digested to a single cell suspension with type V collagenase (Sigma, St. Louis, MO) and DNase I (Roche, Basel, Switzerland) for flow cytometry.

### Cell culture

iBMDMs, RAW264.7 cells, and LLC cells were cultured in Dulbecco’s modified Eagle’s medium (DMEM) (Invitrogen, Carlsbad, CA) containing 10% fetal calf serum (FCS). BMDMs were extracted from the bone marrow of C57BL/6 mice and cultured in DMEM supplemented with 10% FCS and 25 ng/mL murine macrophage colony-stimulating factor (M-CSF) (PeproTech, Rocky Hill, NJ) for 7 days, and flow cytometry analysis was used to detect the stimulation efficiency of BMDMs. The GFP fragment was inserted into the viral vector and then infected into iBMDM to construct a stable cell line for subsequent experiments. In polarization-related experiments, macrophages were stimulated with IFN-γ (20 ng/mL, PeproTech, Rocky Hill, NJ), LPS (50 ng/mL, Sigma, St. Louis, MO) or IL4 (20 ng/mL, PeproTech, Rocky Hill, NJ) for 24 h and then used in follow-up experiments.

### Immunofluorescence

The slides were placed into a 12-well plate and coated with polylysine, and the macrophages were plated on the slide to adhere to the wall, stimulated with IFN-γ, LPS or IL4 for 24 h, and stained with anti-iNOS and anti-ARG1 (CST, Danvers, MA). Mouse subcutaneous tumor tissues were removed and fixed in 4% paraformaldehyde, and then 30% sucrose solution was used to dehydrate them. The tissues were embedded and frozen for sectioning. The sections were stained with anti-F4/80 (Invitrogen, Carlsbad, CA), anti-iNOS or anti-MR and photographed with a fluorescence microscope (M5000, Thermo, Waltham, MA).

### Flow cytometry

Tumor tissue was removed from the subcutaneous skin of mice, cut up, digested with 1 mg/mL collagenase V (Sigma, St. Louis, MO) and 4 mg/mL DNase I (Roche, Basel, Switzerland) at 37°C for 30 min to make a cell suspension, which was filtered through a 70-micron filter membrane and stained with flow cytometry antibody. Dead cells were removed by 7AAD. All the experimental results were analyzed by FACSCalibur and FACSCanto flow cytometry (BD Immunocytometry Systems). Data were processed by FlowJo v10 software (FlowJo, LLC, Ashland, OR).

### Phagocytosis

LLC cell lines were suspended in PBS containing 0.1% serum at a concentration of 10^6^ cells/ml, and the final concentration was 5 nM carboxyfluorescein succinimidyl amino ester (CFSE; MCE, NJ). The cells were stained at room temperature and shielded from light for 7 min. After staining, the same volume of serum was used to terminate the staining, and the stained LLC cells were incubated with macrophages at a ratio of 2:1 for 1 h. The phagocytosis ratio was detected by FACSCanto staining with anti-F4/80 (Invitrogen, Carlsbad, CA). The strength of phagocytosis between different cell lines was compared based on the percentage of double-positive cells in the flow-through results.

### Apoptosis

Macrophages were stimulated with IFN-γ (20 ng/mL, PeproTech, Rocky Hill, NJ), LPS (50 ng/mL, Sigma, St. Louis, MO) or IL4 (20 ng/mL, PeproTech, Rocky Hill, NJ) for 24 h, the supernatant of the stimulated cells was taken. Tumor cells were seeded in 12-well plates, incubated with macrophage supernatant for 48 hours, stained with an Annexin V apoptosis detection kit (Invitrogen, Carlsbad, CA) and detected using FACS Calibur flow cytometry. Effects of macrophage supernatants with different polarization states on early apoptosis, mid-apoptosis, and late apoptosis of tumor cells analyzed by flow data analysis.

### Cell proliferation

The LLC cell line was seeded in 12-well plates, macrophage supernatants of different stimulation states were taken and incubated with tumor cells in LLC for 24 h. After fixation with 4% paraformaldehyde and staining with crystal violet (Kehao, Xi’an, China), the supernatants were washed with PBS 3 times and resuspended with acetic acid, the absorbance of the liquids was measured by an enzyme marker, and the effect of macrophage supernatants of each polarization state on the proliferation of tumor cells was compared based on the strength of the absorbance.

### Cell migration

A marker was used to draw three lines on the back of the 12-well plate, and the LLC cells were inoculated into the 12-well plate. When the cells grew to 80%, the tip of the gun was used to draw a straight line perpendicular to the three lines, macrophage supernatants of different polarization states were added, photos of the scratch were taken at the intersection of the 3 straight lines according to different times, and the cell migration area was counted.

### RT−PCR

Total cellular and tissue RNA was extracted with TRIzol (Invitrogen, Carlsbad, CA) reagent according to the instructions, and the concentration of the extracted RNA was determined and then transcribed into CDNA using a reverse transcription kit (Yeasen, Shanghai, China). SYBR Premix EX Taq (Yeasen, Shanghai, China) was added to the system according to its instructions, and real-time quantitative PCR was performed by QuantStudio.

### Statistics

All experimental data were statistically processed by GraphPad Prism 5 software, and unpaired Student’s t tests or one-way ANOVA was used for comparison. When the data results were expressed as P<0.05, they were considered statistically significant.

## Results

### iBMDMs exhibit the similar cellular characteristics of primary macrophage

**Figure 1:**
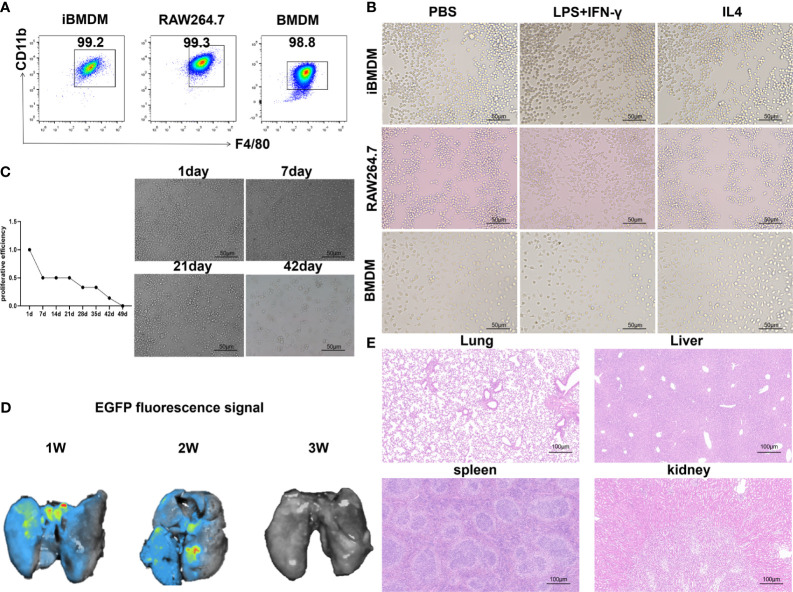
iBMDMs exhibit the similar cellular characteristics of primary macrophage. **(A)** Flow cytometry was used to detect macrophage-specific markers after staining with F4/80 and CD11b for iBMDMs, RAW264.7 cells, and BMDMs. **(B)** Using PBS, LPS+IFN-γ and IL-4 stimulated iBMDMs, RAW264.7 cells, and BMDMs, morphological changes in macrophages were observed after polarization stimulation using an inverted microscope. **(C)** Pass iBMDMs at a ratio of 1:10. An inverted microscope was used to take photos and observe morphological changes. Determine cell proliferation activity *in vitro* based on the length of its passage cycle. **(D)** iBMDMs were injected into mice, and fluorescence signal expression at different times was detected to determine the survival time of iBMDMs in mice. **(E)** Injection of ibmdm into mice and detection of the biological safety of iBMDMs in mice by HE staining of mouse tissues.

CD11b and F4/80 are specific markers for macrophages (34). To identify the macrophage characteristics of iBMDMs, we performed FACS analysis by anti-F4/80 and anti-CD11b staining. As expected, iBMDMs, BMDMs and RAW264.7 cells shared similar macrophage biomarker expression patterns (**Figure 1A**). For further morphological comparison, the three kinds of macrophages were stimulated with different cytokines for observation by microscopy. The M1-polarized BMDMs had some appearing cogwheel, while M2-polarized BMDMs had longer pseudopodia compared with controls. The RAW264.7 cells protruded more pseudopodia after polarization stimulation. In contrast to polarized BMDMs or RAW264.7 cells, iBMDMs presented polygon or round shapes regardless of the cytokines added (**Figure 1B**).

Next, we verified the survival and biosafety of iBMDMs *in vivo* and *in vitro*. *In vitro*, we cultured the cells for a long time and passaged them at a ratio of 1:10 each time. According to the passage cycle, the proliferation activity of the cells was measured. The results showed that the proliferation efficiency of iBMDMs began to slow down after 3 weeks of *in vitro* culture and was almost quiescent after 6 weeks (**Figure 1C**). Similarly, we validated the survival cycle of iBMDMs in mice. iBMDMs survived for 3 weeks, but the number of surviving iBMDMs gradually decreased over time (**Figure 1D**). The results suggested that iBMDMs possessed a long-term lifespan but no immortalization capacity both *in vivo* and *in vitro*, which provided feasibility for iBMDM-based cell therapy. To ensure the biosafety of iBMDMs, we carried out HE staining by using sections of different tissues from iBMDM reinfused mice. The results showed that iBMDMs were nontoxic to mouse tissue and can be used for subsequent treatment in mice (**Figure 1E**). The above data suggested that iBMDMs maintained rapid proliferation and long-term lifespan, indicating potential cell sources for immunotherapy.

### Normal polarization response is possible with iBMDMs

**Figure 2:**
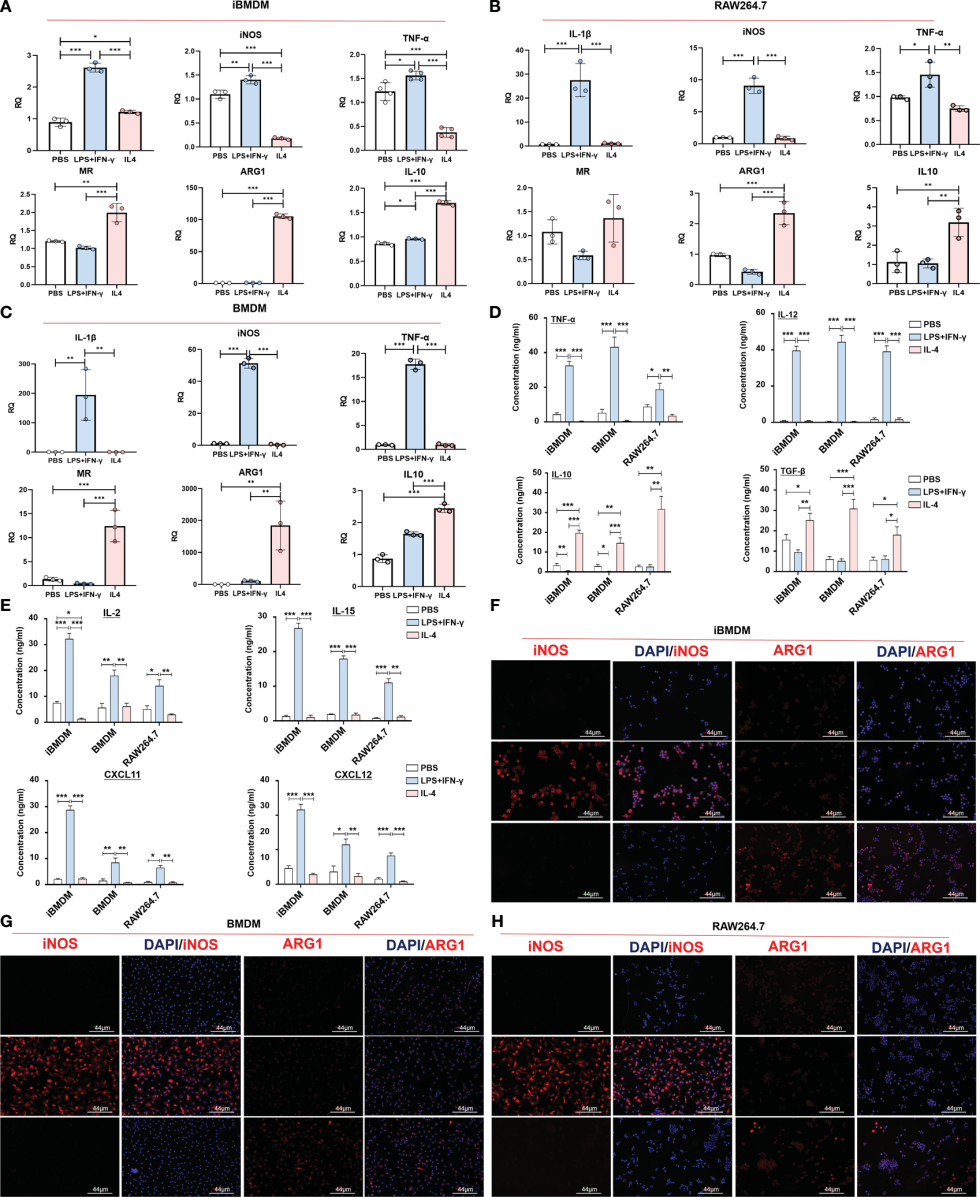
Normal polarization response is possible with iBMDM. **(A–G)** iBMDMs, RAW264.7 cells and BMDMs were treated with PBS, LPS+IFN-γ and IL4 for 24 hours. **(A–C)** QPCR was used to detect the expression of M1 polarization markers (IL-1β and iNOS) and M2 polarization markers (MR and ARG1) in iBMDMs, RAW264.7 cells, and BMDMs under different stimuli. **(D, E)** Macrophage supernatant was collected under different stimuli, and ELISA was used to detect the expression of M1 markers (TNF-α and IL-12), M2 markers (IL10 and TGF-β), T cell-associated functional factors (IL2 and IL15) and chemokines (CXCL11 and CXCL12) in the macrophage supernatant. **(F–H)** Anti-iNOS and anti-ARG1 were used as primary antibodies for immunofluorescence staining and to detect the expression of markers after macrophage polarization. Bars, mean ± SEM; *P < 0.05; **P < 0.01; ***P < 0.001.

Phenotypic alterations in iBMDMs were assessed by qPCR detection of specific M1 (IL-1β and iNOS) and M2 (Arg1 and MR) polarization biomarkers. The results revealed that after LPS+INF-γ treatment, the levels of M1 genes were specifically increased in all three types of macrophages. In addition, the expression of M2 polarization markers was obviously increased in IL-4-treated macrophages. It should be noted that although the iBMDMs presented a similar polarization response to primary BMDMs or macrophage lines, the mRNA elevations of all M1-specific biomarkers (IL-1β, iNOS and TNF-α) were mild in iBMDMs compared with RAW264.7 cells or BMDMs. Compared to RAW264.7, there was no significant difference in the M2 biomarkers of iBMDM, but compared to BMDM, the increase of iBMDM was milder under IL4 stimulation (**Figures 2A–C**). The ELISA assay also demonstrate that iBMDM can be polarized with different stimuli, and the results indicate that M1-iBMDMs produced much higher levels of proinflammatory cytokines (TNF-α and IL-12) and lower levels of anti-inflammatory factors (IL-10 and TGF-β). Interestingly, the differences in secreted proteins between the three kinds of macrophages were not particularly significant. Under both M1- and M2-polarization conditions, iBMDMs presented the same level of inflammatory response as BMDMs (**Figure 2D**). M1-type macrophages have elevated aerobic glycolysis and produce inducible nitric oxide synthase (iNOS), which is associated with antitumor and anti-infection immunity (35). ARG1 is an enzyme involved in arginine metabolism and generation in macrophages that leads to T-cell exhaustion and functional repression (36). iNOS and ARG1 are essential markers of M1 polarization and M2 polarization, respectively. To further observe the expression of polarization markers in different cell lines, three kinds of macrophages were stimulated and stained with anti-iNOS and anti-ARG1. Meanwhile, we determined the effect of surpernatant from different macrophages on T cell activation. The results of ELISA assay suggested that M1-iBMDMs secreted higher levels of chemokines CXCL11 and CXCL12 to promote T-cell recruitment. The productions of T cells activation cytokines, including IL2 and IL15, were also increased in M1-iBMDMs (**Figure 2E**). The immunofluorescence results showed a similar conclusion that M1-iBMDMs expressed higher levels of iNOS, while M2-iBMDMs exhibited advantages in ARG1 expression, which was even more obvious than that of BMDMs and RAW264.7 cells (**Figures 2F–H**). These data suggested that iBMDMs perform a similar polarization response as other macrophage sources.

### iBMDMs strongly phagocytose tumor cells

**Figure 3:**
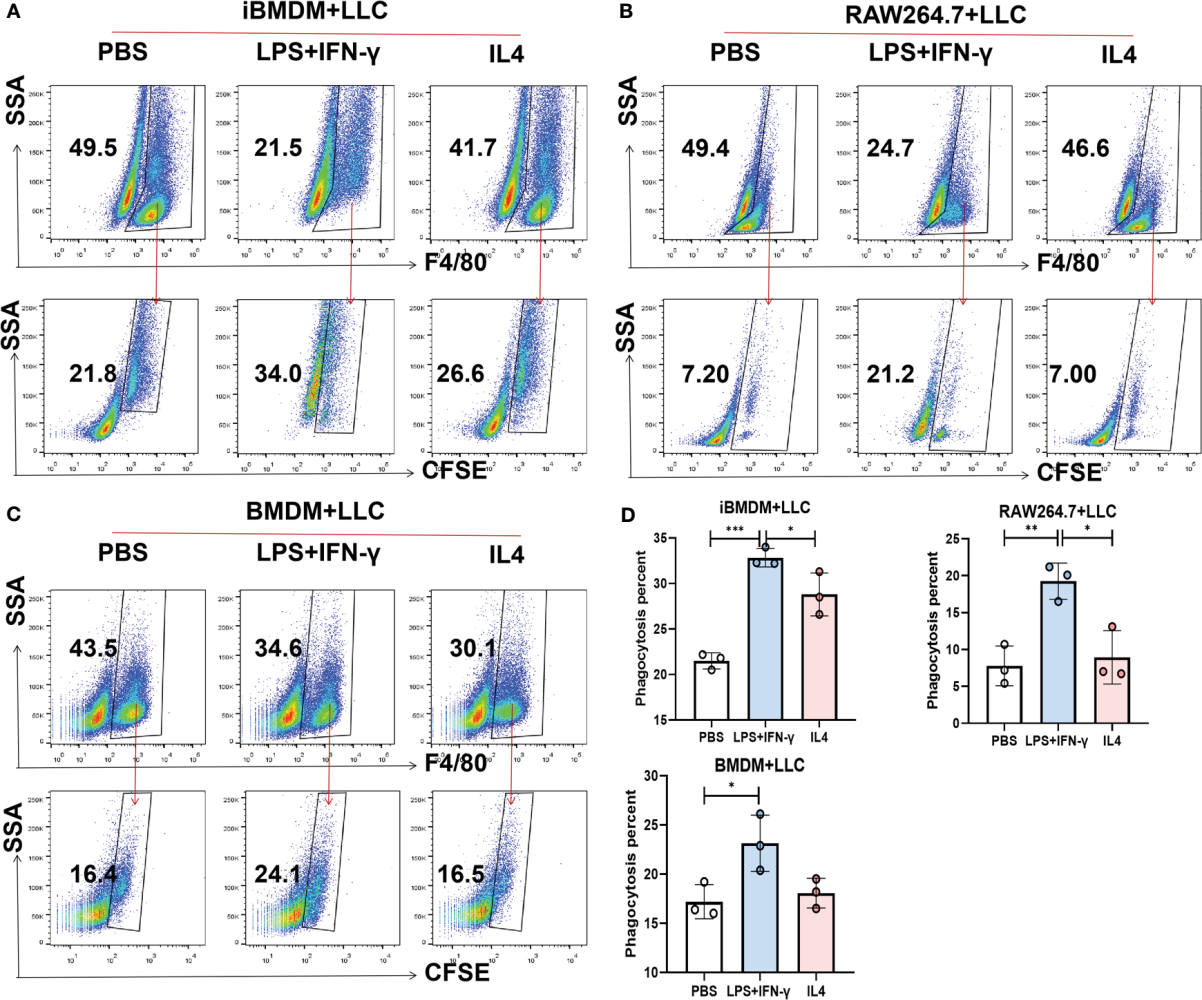
iBMDMs strongly phagocytose tumor cells. **(A-D)** iBMDMs, RAW264.7 cells and BMDMs were treated with PBS, LPS+IFN-γ and IL4 for 24 hours. Differently treated iBMDMs, RAW264.7 cells, and BMDMs were coincubated with LLCs at a ratio of 1:2 for one hour, and the phagocytosis ability of macrophages was detected by flow cytometry. Bars, mean ± SEM; *P < 0.05; **P < 0.01; ***P < 0.001.

Next, we explored the phagocytosis function of macrophages, which plays a key role in tumor killing and pathogen removal (37). To examine the differences in phagocytosis among the three kinds of macrophages, iBMDMs, RAW264.7 cells and BMDMs were stimulated with polarization factors for 24 h and then cocultured with CFSE-stained LLC cells at a ratio of 1:2. Two hours later, the phagocytosis capacity was evaluated by calculating the proportion of macrophages swallowing tumor cells (F4/80^+^CFSE^+^) using flow cytometry. It should be noted that the phagocytic ability of M1-BMDMs was elevated 10-fold compared with that of quiescent BMDMs, which is equivalent to the response level of RAW264.7 cells. However, after LPS+INF-γ treatment, iBMDMs presented much stronger phagocytic enhancement. The engulfment rate of M1-loaded iBMDMs was almost 20 times that of quiescent iBMDMs (**Figures 3A–D**). In summary, M1-iBMDMs exhibited strong phagocytosis, which was stronger than that of BMDMs and RAW264.7 cells, and the results further demonstrated that immortalized BMDMs could interact well with tumor cells.

### The paracrine of iBMDMs inhibits proliferation and promotes apoptosis of tumor cells

**Figure 4:**
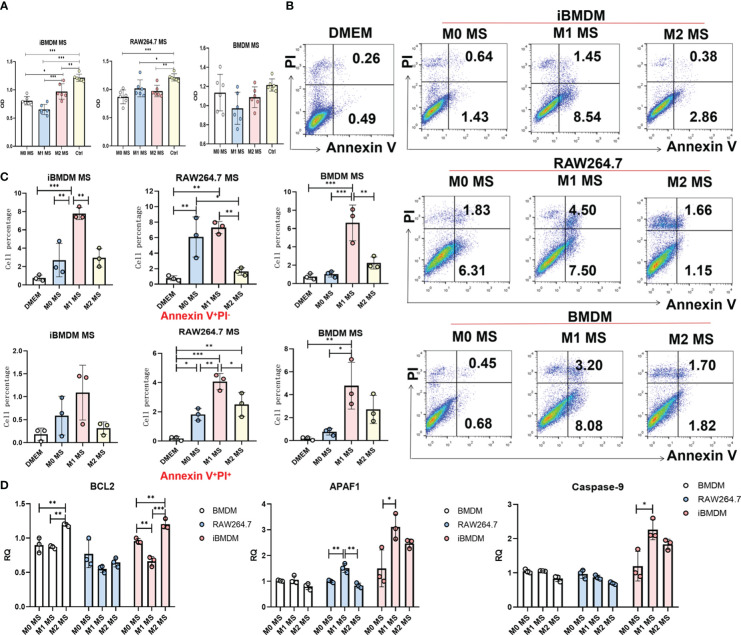
The paracrine of iBMDMs inhibits proliferation and promotes apoptosis of tumor cells. **(A–D)** iBMDMs, RAW264.7 cells and BMDMs were treated with PBS, LPS+IFN-γ and IL4 for 24 hours. **(A)** iBMDMs, RAW264.7 cells and BMDM supernatants were coincubated with LLCs in a 96-well plate for 24 h, and the absorbance was measured by an enzyme labeling instrument after crystal violet staining to detect the effect of macrophage supernatants on the proliferation of tumor cells. **(B, C)** iBMDM, RAW264.7, and BMDM supernatants were coincubated with LLCs for 48 h, and apoptosis of tumor cells was detected by flow cytometry. Bars, mean ± SEM; *P < 0.05; **P < 0.01; ***P < 0.001. **(D)** Detection of apoptosis-related genes (BCL2, APAF1, Caspase-9) in LLC after macrophage supernatant treatment using QPCR.

In addition to direct phagocytosis to inhibit tumor progression, macrophages can also modulate tumor cell migration, proliferation and apoptosis by secreting multiple cytokines and inflammatory mediators. To further verify the macrophage function of iBMDMs, we incubated LLC cells in the supernatant of macrophages with different treatments for 24 hours and determined cell proliferation by measuring absorbance after staining with crystal violet. As expected, the supernatant of all three groups of macrophages exhibited the inhibition of tumor cell proliferation, especially that of M1 macrophages. The results were consistent with previous results showing that M1-type macrophages have the ability to inhibit tumor growth. Interestingly, compared with RAW264.7 cells and BMDMs, iBMDMs showed a significant decrease in tumor cell proliferation after M1 polarization. This result proved that the iBMDMs had a stronger antitumor function (**Figure 4A**).

Furthermore, we cocultured LLC cells with macrophage supernatant for 48 hours and detected LLC apoptosis by using Annexin V/PI staining. We found that the control group without co-culture of macrophages showed that tumor cells almost did not undergo apoptosis in the absence of co-culture. And the supernatant of M1 macrophages promoted tumor cell apoptosis more obviously than PBS-treated macrophages. In addition, although the iBMDM supernatant displayed a certain ability to induce tumor cell apoptosis, the percentage of late apoptotic cells was much lower than that of LLC cells incubated with supernatant from BMDMs or RAW264.7 cells (**Figures 4B, C**). We speculate that the factors secreted by iBMDMs mainly influence the early stage of tumor cell apoptosis. In addition, we have tested the apoptosis function of tumor cells incubating with supernatant of different macrophages by qRT-PCR. The results showed that supernatant of M1-iBMDMs promoted the expression of apoptosis associated genes, including APAF1 and Caspase-9 in tumor cells, and decreased the level of protective molecule BCL2 (**Figure 4D**). Considered that TNF-α, which was increased in M1-iBMDM (**Figure 2D**), possesses the effect of promoting tumor cells apoptosis, it is reasonable that iBMDM induced apoptosis of tumor cells through cytotoxic cytokine such as TNF-α. In summary, the secreted component of iBMDMs had obvious effects on inhibiting tumor cell proliferation and promoting apoptosis, which indicated that iBMDMs could also repress tumor growth indirectly.

### iBMDMs repress tumor cell migration via inhibiting EMT progress

**Figure 5:**
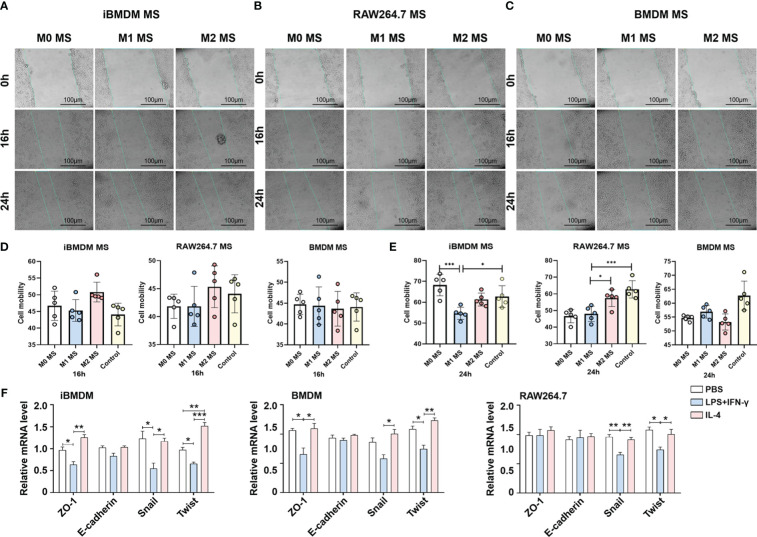
iBMDMs repress tumor cell migration via inhibiting EMT progress. **(A–C)** PBS, LPS+IFN-γ, IL4-stimulated iBMDMs, RAW264.7 cells, and BMDMs for 24 h. iBMDMs, RAW264.7 cells, and BMDM supernatants with different polarization states were coincubated with LLCs, and the effect of macrophage supernatants on tumor cell migration was detected by scratch. **(D, E)** ImageJ software was used to process the scratch results and generate statistics on the processing results. **(F)** iBMDMs, RAW264.7 cells, and BMDM supernatants were coincubated with tumor cells for 24 h, and the expression of EMT (epithelial-mesenchymal transition)-related genes was detected by QPCR. Bars, mean ± SEM; *P < 0.05; **P < 0.01; ***P < 0.001.

Modulating tumor cell invasion and participating in the formation of migrated units are important functions of tumor-associated macrophages. The wound healing assay was performed by incubating tumor cells with macrophage supernatants to test the cell motion at different time periods. The results showed that the modulation effect was not obvious after 16 h of incubation. However, tumor migration was significantly inhibited by M1 macrophage supernatant after 24 h treatment. As expected, iBMDMs, especially M1-iBMDMs, presented the most remarkable inhibitory effect (**Figures 5A–E**).

Multiple factors can influence tumor cell infiltration and metastasis. To investigate the mechanisms by which macrophage paracrine signaling affects LLC mobility, we cocultured macrophage supernatants with LLC cells for 24 h and then detected the expression of EMT (epithelial-mesenchymal transition) -related genes, which are responsible for tumor cell migration to some extent (38). The data suggested that iBMDMs exhibited notable repressive effects on LLC EMT. After administration of M1-iBMDM supernatant, the expression of the tight junction-related membrane protein ZO-1 and the EMT essential transcription factors Snail1 and Twist was greatly reduced, which was superior to the other two types of macrophages (**Figure 5F**). The above results demonstrated that the paracrine pathway of iBMDMs plays a significant role in tumor cell EMT progression and migration regulation.

### M1-polarized iBMDMs rather than primary BMDMs repress tumor growth *in vivo*

**Figure 6:**
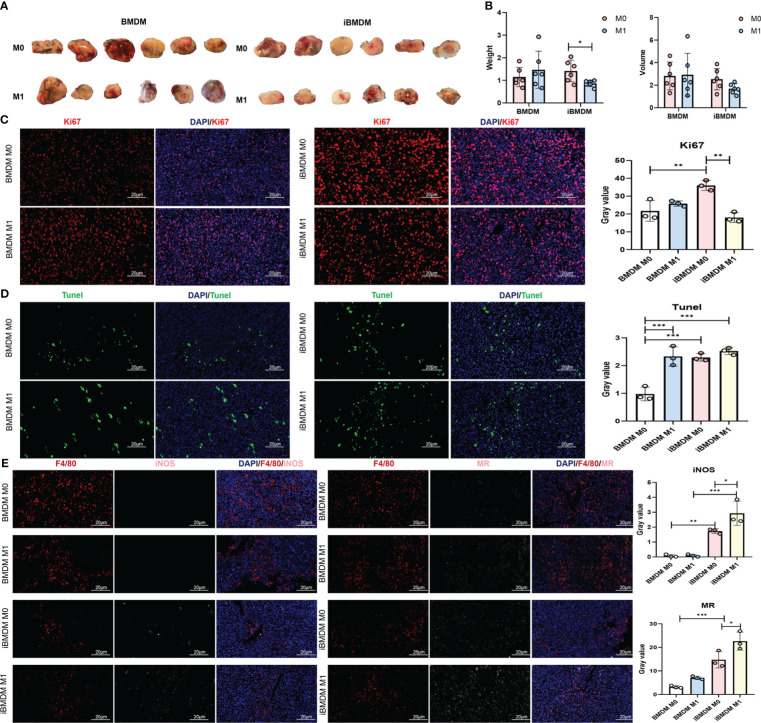
M1-polarized iBMDMs rather than primary BMDMs repress tumor growth *in vivo*. **(A, B)** iBMDMs and BMDMs were stimulated with PBS or LPS + IFN-γ for 24 h, mixed with LLC at a ratio of 1:5, and inoculated subcutaneously, and the size and weight of the tumors were measured and compared after 3 weeks. **(C, D)** Immobilization, embedding, and sectioning of subcutaneous tumor tissue were performed using Ki67 and TUNEL staining. Fluorescence microscopy was used to detect the expression of Ki67 and TUNEL in tumor tissue, and the effects of the two types of macrophages on tumor cell proliferation and apoptosis in the tumor microenvironment were compared. **(E)** Perform F4/80, iNOS, and MR staining on subcutaneous tumor sections. Fluorescence microscopy photography was used to detect the impact of the two types of macrophages on the tumor microenvironment in tumor tissue. Bars, mean ± SEM; *P < 0.05; **P < 0.01; ***P < 0.001.

Previous experiments have demonstrated the macrophage characteristics and antitumor functions of iBMDMs *in vitro*. To verify the phenotypes and effects of iBMDMs during tumor progression, we stimulated EGFP-modified BMDMs or iBMDMs into M1 polarization and mixed them with LLC at a ratio of 1:5 to inoculate them subcutaneously on the backs of mice. The tumor size and weight were monitored after 3 weeks. The tumor volume and weight in the M1-iBMDM group were smaller than those in the M0-iBMDM group, which had an inhibitory effect on tumor growth. Compared with BMDMs, M1-BMDMs did not have a significant inhibitory effect and even showed an upward trend (**Figures 6A, B**). The results showed that the tumor size and weight of the iBMDM group treated with M1 polarization were significantly decreased compared with those of the control group (M0 group) (**Figure 6A**). Meanwhile, the changes in tumor size and weight in the BMDM infusion groups were not obvious and were even increased in the M1-BMDM treatment group (**Figure 6B**). Ki67 and TUNEL staining was also performed using tumor sections. The Ki67 staining data suggested that there was no significant change between the M1-BMDM and M0-BMDM groups. Meanwhile, M1-iBMDM treatment inhibited tumor cell proliferation and reduced tumor malignancy. (**Figure 6C**). TUNEL staining showed that M0-iBMDMs significantly promoted tumor cell apoptosis compared with M0-BMDMs. Interestingly, there was no significant difference between M1-iBMDMs and M1-BMDMs (**Figure 6D**). These results indicated that M1-polarized iBMDMs possessed significant antitumor activity *in vivo* by modulating the malignant biological behaviors of tumor cells.

Next, we examined the expression of representative molecular markers of different polarization phenotypes and macrophage function with tumor sections. The immunofluorescence results showed that there were significantly more M2-type macrophages (MR^+^ F4/80^+^) than M1-type macrophages (iNOS^+^ F4/80^+^) in the tumor tissues of both the iBMDM and BMDM groups. However, regardless of M0- and M1-polarized BMDM therapy, few iNOS-positive macrophages were detected after three weeks, suggesting that BMDM infusion did not domesticate the recruited macrophages. This might be because the survival time of infused BMDMs was too short to exert the immune regulatory function completely. In contrast, iBMDM infusion stimulated more M1-like TAMs to improve the immune microenvironment and repress tumor growth. Especially after the infusion of M1-type iBMDMs, the number of iNOS-positive TAMs increased significantly (**Figure 6E**). Most infused macrophages could not last their lifespan to 3 weeks in tumors. Even long-term iBMDMs could not be detected in the infused tissue *in vivo* (**Figure 1**). Obviously, the infused iBMDMs had a profound and sustained impact on the endogenous recruited macrophages and tumor microenvironment. The effect of switching TAMs into the M1-like phenotype might be the reason why M1-iBMDM therapy could inhibit tumor development.

### M1-polarized iBMDMs domesticate self-recruited TAMs and improve the tumor microenvironment

**Figure 7:**
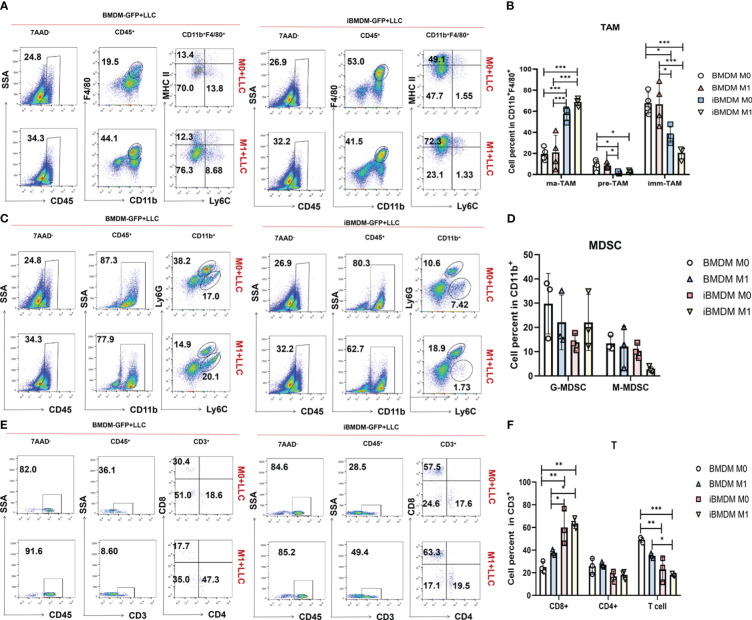
M1-polarized iBMDMs domesticate self-recruited TAMs and improve the tumor microenvironment. **(A-F)** PBS, LPS+IFN-γ-stimulated iBMDMs, and BMDMs for 24 h were mixed with LLC at a ratio of 1:5 and then inoculated subcutaneously. After 3 weeks of tumor inoculation, flow cytometry was used to evaluate the tumor immune microenvironment of mice with different treatments, and the percentages of TAMs **(A, B)**, MDSCs **(C, D)**, and T cells **(E, F)** in the tumor immune microenvironment were detected and analyzed. Bars, mean ± SEM; *P < 0.05; **P < 0.01; ***P < 0.001.

To further investigate the impact of iBMDMs on the tumor microenvironment, we digested the tumor tissues into single-cell suspensions for further FACS analysis. TAMs were classified into three subgroups based on Ly6C and major histocompatibility complex class II (MHCII class) expression: mature TAMs (ma-TAMs) (Ly6C^-^MHCII^+^), immature TAMs (imm-TAMs) (Ly6C^-^MHCII^-^), and TAM precursors (pre-TAMs) (Ly6C^+^MHCII^-^) (39, 40). Many studies have demonstrated that mature TAMs highly express M1 polarization-related markers and exert antitumor functions. Compared to that in the BMDM group, the proportion of mature TAMs in the iBMDM group was significantly increased, while the proportion of immature TAMs and TAM precursors was significantly decreased. Similarly, the M1 iBMDM group had a significantly higher population of Ly6C^-^MHCII^+^ ma-TAMs than the M0-iBMDM treatment group, while BMDMs did not have this result (**Figures 7A, B**).

G-MDSCs and M-MDSCs play an important role in the tumor microenvironment. In tumor-related myeloid cells (CD11b^+^), the proportions of G-MDSCs and M-MDSCs in the iBMDM group were lower than those in the BMDM group. iBMDMs can reduce G-MDSCs and M-MDSCs in the tumor microenvironment, improve the tumor microenvironment, and inhibit tumor growth. Moreover, M1 iBMDMs had a more significant inhibitory effect on M-MDSCs (**Figures 7C, D**). In addition, iBMDMs can promote the recruitment of CD8^+^ T cells in tumors, increase the direct killing of tumor cells in the microenvironment, and further improve the microenvironment of tumors (**Figures 7E, F**). Our *in vitro* experiment results had demonstrated that the paracrine pathway of M1-iBMDM could promote the recruitment and activation of T cells (**Figure 2E**), which could explained the promotion of T cell population. In summary, iBMDMs not only have excellent macrophage function *in vitro* but also improve the tumor microenvironment *in vivo*, recruiting endogenous macrophages to exhibit an antitumor phenotype while reducing the proliferation of various tumor-promoting immune cells and increasing the number of antitumor CD8^+^ T cells, thus inhibiting tumor growth. *In vivo*, the therapeutic effect of iBMDMs is superior to that of BMDMs.

## Discussion

Macrophages are considered as the key immune regulator during tumor initiation and development. Macrophages not only contribute to the recruitment and activation of immune cells in the tumor microenvironment (TME) but also play an important role in tumor cell metastasis (41). Targeting tumor-associated macrophages (TAMs) is currently considered a promising strategy for combating cancer (42). How to polarize TAMs to an antitumor state without affecting macrophage activity is of great research significance to reduce tumor growth and metastasis. The current understanding of the mechanisms involved in controlling the cancer and metastatic cascade response remains limited. Studies of mechanisms that regulate innate immune activation require *in vitro* cellular experiments or *in vivo* therapeutic validation. Currently, the most common used cell resources for macrophage research are BMDMs and RAW264.7 cells (28). BMDMs, as primary bone marrow-derived cells, need to be obtained from the bone marrow followed by stimulation and cultivation with different cytokines, which requires much more time and cost. Meanwhile, the short survival time of BMDMs is not suitable for establishing a stable transduced cell line, which limits their application for *in vivo* transfusion therapy (43). In contrast, RAW264.7 is a type of peritoneal macrophage from confluent mice that has immunogenicity and tumor-promoting properties and is not suitable for *in vivo* reperfusion therapy. Immortalized macrophages may help meet the current needs of reducing macrophage research costs and establishing stable and transmissible cell lines.

This study introduces the iBMDM cell line constructed by Elisabetta Blasi et al. in 1986 (29) and compares it with BMDM and RAW264.7 cell lines in terms of phenotype, characteristics, polarization detection, and *in vitro* and *in vivo* antitumor functions, proving the feasibility of using iBMDM cell lines as macrophages for research within a certain range. Previous studies have not systematically compared the differences in function and characteristics between immortalized macrophages and nonimmortalized cells and explored their advantages and disadvantages as macrophage therapy. This experiment validated the relative safety and effectiveness of using iBMDMs as a macrophage therapy resource. We also investigated macrophage polarization regulation and tumor microenvironment domestication reversal, especially the influence on endogenously recruited macrophages. Our data indicate that the iBMDM cell line is actually not immortalized but possesses a relatively longer survival time both *in vitro* and *in vivo*. The long-term lifespan of iBMDMs provides more possibilities for cell therapy while ensuring biological safety because they do not remain in the body after reinfusion. In addition, we also demonstrated that although iBMDMs exhibited similar characteristics as other macrophages (BMDMs and RAW264.7 cells), certain differences were found among different macrophage sources in the expression levels of polarization markers and their *in vitro* and *in vivo* antitumor functions. It should be noted that iBMDMs were demonstrated to exhibit a superior ability to improve the tumor microenvironment and repress tumor development compared to BMDMs. Our data indicated that iBMDMs facilitated T cell recruitment and activation via chemokines and cytokines secretion both *in vitro* and *in vivo* (**Figures 2E, F**). However, this does not fully represent the remodeling of T cell function. The role of iBMDMs on T cells and microenvironment needs to be further investigated.

In conclusion, our data indicate that these long-term iBMDMs possess macrophage characteristics and functions and are superior to other macrophages in some aspects. It can be used for *in vivo* and *in vitro* experiments on macrophages and is expected to serve as a cell resource for macrophage reinfusion therapy. Moreover, iBMDMs could be further modified with genetic editing. The edited iBMDMs presented a more stable phenotype and stronger antitumor functions. Therefore, iBMDMs have great potential for application in immune cell therapy. We also hope to further investigate the molecular mechanisms of the differences between BMDMs and iBMDMs. To advance research on macrophage therapy and its clinical application as soon as possible, more comprehensive and in-depth research needs to be implemented to identify the key molecules involved in phenotypic and functional changes. The main difficulties limiting iBMDMs-based therapy to human patients is that no human immortalized BMDMs could be used for modification or gene-editing for immune-therapies. The further investigating is required to solve how to immortalize human-derived BMDMs. Meanwhile, the quantitative systems pharmacology modeling has realized the accurate prediction of tumor therapy and the evaluation of immunotherapy effects by combining pharmacokinetics, pharmacodynamics and disease progression. The model has greatly improved the research and development efficiency of cellular immunotherapy and combination drugs (44, 45). It provides an excellent theoretical basis for the promotion of clinical immunotherapy. The use of quantitative systems pharmacology modeling could help the research focus on modified-iBMDMs and human immortalized-macrophages for immunotherapy in clinic. The revelation of the immortalization characteristics of macrophages also provides a reference for the immortalization of other immune cells (such as NK cells, dendritic cells, and T cells). and lays a foundation for further improving tumor immunotherapy.

## Data availability statement

The original contributions presented in the study are included in the article/supplementary material. Further inquiries can be directed to the corresponding authors.

## Ethics statement

The animal study was approved by Animal Experiment Administration Committee of the Fourth Military Medical University. The study was conducted in accordance with the local legislation and institutional requirements.

## Author contributions

DKX: Conceptualization, Data curation, Formal analysis, Investigation, Methodology, Project administration, Validation, Visualization, Writing - original draft, Writing - review & editing. JY: Formal analysis, Investigation, Methodology, Validation, Writing - original draft. PHL: Conceptualization, Formal analysis, Investigation, Validation, Writing - original draft. YWZ: Formal analysis, Investigation, Methodology, Validation, Writing - original draft. JNC: Formal analysis, Investigation, Writing - original draft. XLC: Methodology, Funding acquisition, Writing - original draft. SLC: Investigation, Methodology, Validation, Writing - original draft. YMC: Formal analysis, Investigation, Visualization, Writing - original draft. YFH: Formal analysis, Validation, Writing - original draft. LW: Data curation, Methodology, Validation, Writing - original draft. ZHW: Investigation, Visualization, Writing - original draft. RQ: Formal analysis, Validation, Writing - original draft. JMG: Investigation, Visualization, Writing - original draft. HY: Investigation, Writing - original draft. LW: Formal analysis, Validation, Writing - original draft. ZYL: Investigation, Methodology, Writing - original draft. HH: Conceptualization, Supervision, Writing - review & editing. HYQ: Conceptualization, Funding acquisition, Methodology, Project administration, Resources, Supervision, Writing - review & editing. JLZ: Conceptualization, Funding acquisition, Methodology, Project administration, Resources, Supervision, Writing - review & editing.

## References

[1] Martinez FO Gordon S Locati M Mantovani A. Transcriptional profiling of the human monocyte-to-macrophage differentiation and polarization: new molecules and patterns of gene expression. J Immunol. (2006) 177:7303–11. doi: 10.4049/jimmunol.177.10.7303 17082649

[2] Varol C Mildner A Jung S. Macrophages: development and tissue specialization. Annu Rev Immunol. (2015) 33:643–75. doi: 10.1146/annurev-immunol-032414-112220 25861979

[3] Anderson NR Minutolo NG Gill S Klichinsky M. Macrophage-based approaches for cancer immunotherapy. Cancer Res. (2021) 81:1201–8. doi: 10.1158/0008-5472.CAN-20-2990 33203697

[4] Biswas SK Allavena P Mantovani A. Tumor-associated macrophages: functional diversity, clinical significance, and open questions. Semin Immunopathol. (2013) 35:585–600. doi: 10.1007/s00281-013-0367-7 23657835

[5] De Palma M Lewis CE. Macrophage regulation of tumor responses to anticancer therapies. Cancer Cell. (2013) 23:277–86. doi: 10.1016/j.ccr.2013.02.013 23518347

[6] Cutolo M Campitiello R Gotelli E Soldano S. The role of M1/M2 macrophage polarization in rheumatoid arthritis synovitis. Front Immunol. (2022) 13:867260. doi: 10.3389/fimmu.2022.867260 35663975 PMC9161083

[7] Epelman S Lavine KJ Randolph GJ. Origin and functions of tissue macrophages. Immunity. (2014) 41:21–35. doi: 10.1016/j.immuni.2014.06.013 25035951 PMC4470379

[8] Mantovani A Allavena P Marchesi F Garlanda C. Macrophages as tools and targets in cancer therapy. Nat Rev Drug Discovery. (2022) 21:799–820. doi: 10.1038/s41573-022-00520-5 35974096 PMC9380983

[9] Yunna C Mengru H Lei W Weidong C. Macrophage M1/M2 polarization. Eur J Pharmacol. (2020) 877:173090. doi: 10.1016/j.ejphar.2020.173090 32234529

[10] Wu K Lin K Li X Yuan X Xu P Ni P. Redefining tumor-associated macrophage subpopulations and functions in the tumor microenvironment. Front Immunol. (2020) 11:1731. doi: 10.3389/fimmu.2020.01731 32849616 PMC7417513

[11] Huang F Zhao JL Wang L Gao CC Liang SQ An DJ. miR-148a-3p mediates notch signaling to promote the differentiation and M1 activation of macrophages. Front Immunol. (2017) 8:1327. doi: 10.3389/fimmu.2017.01327 29085372 PMC5650608

[12] Ren K Li S Liang S Fan F Lu J Wei T. Notch signaling dependent monocyte conversion alleviates immune-mediated neuropathies by regulating RBP-J/NR4A1 axis. J Autoimmun. (2022) 133:102945. doi: 10.1016/j.jaut.2022.102945 36356552

[13] Li SZ Ren KX Zhao J Wu S Li J Zang J. miR-139/PDE2A-Notch1 feedback circuit represses stemness of gliomas by inhibiting Wnt/beta-catenin signaling. Int J Biol Sci. (2021) 17:3508–21. doi: 10.7150/ijbs.62858 PMC8416740 34512162

[14] Wang L Hu YY Zhao JL Huang F Liang SQ Dong L. Targeted delivery of miR-99b reprograms tumor-associated macrophage phenotype leading to tumor regression. J Immunother Cancer. (2020) 8:e000517. doi: 10.1136/jitc-2019-000517 32948650 PMC7511616

[15] Zhao J Li H Zhao S Wang E Zhu J Feng D. Epigenetic silencing of miR-144/451a cluster contributes to HCC progression via paracrine HGF/MIF-mediated TAM remodeling. Mol Cancer. (2021) 20:46. doi: 10.1186/s12943-021-01343-5 33658044 PMC7927270

[16] Zhao JL Huang F He F Gao CC Liang SQ Ma PF. Forced activation of notch in macrophages represses tumor growth by upregulating miR-125a and disabling tumor-associated macrophages. Cancer Res. (2016) 76:1403–15. doi: 10.1158/0008-5472.CAN-15-2019 26759236

[17] Cassetta L Pollard JW. Pollard, Targeting macrophages: therapeutic approaches in cancer. Nat Rev Drug Discovery. (2018) 17:887–904. doi: 10.1038/nrd.2018.169 30361552

[18] Xiao M Bian Q Lao Y Yi J Sun X Sun X. SENP3 loss promotes M2 macrophage polarization and breast cancer progression. Mol Oncol. (2022) 16:1026–44. doi: 10.1002/1878-0261.12967 PMC8847990 33932085

[19] Toda G Yamauchi T Kadowaki T Ueki K. Preparation and culture of bone marrow-derived macrophages from mice for functional analysis. STAR Protoc. (2021) 2:100246. doi: 10.1016/j.xpro.2020.100246 33458708 PMC7797923

[20] Xue D Lu S Zhang H Zhang L Dai Z Kaufman DS. Induced pluripotent stem cell-derived engineered T cells, natural killer cells, macrophages, and dendritic cells in immunotherapy. Trends Biotechnol. (2023) 41:907–22. doi: 10.1016/j.tibtech.2023.02.003 36858941

[21] Takata K Kozaki T Lee CZW Thion MS Otsuka M Lim S. Induced-pluripotent-stem-cell-derived primitive macrophages provide a platform for modeling tissue-resident macrophage differentiation and function. Immunity. (2017) 47:183–198.e6. doi: 10.1016/j.immuni.2017.06.017 28723550

[22] Zhang L Tian L Dai X Yu H Wang J Lei A. Pluripotent stem cell-derived CAR-macrophage cells with antigen-dependent anti-cancer cell functions. J Hematol Oncol. (2020) 13:153. doi: 10.1186/s13045-020-00983-2 33176869 PMC7656711

[23] Li P Hao Z Wu J Ma C Xu Y Li J. Comparative proteomic analysis of polarized human THP-1 and mouse RAW264.7 macrophages. Front Immunol. (2021) 12:700009. doi: 10.3389/fimmu.2021.700009 34267761 PMC8276023

[24] Jin Z Zhu Z Zhang W Liu L Tang M Li D. Effects of TRIM59 on RAW264.7 macrophage gene expression and function. Immunobiology. (2021) 226:152109. doi: 10.1016/j.imbio.2021.152109 34252840

[25] Zeng XZ He LG Wang S Wang K Zhang YY Tao L. Aconine inhibits RANKL-induced osteoclast differentiation in RAW264.7 cells by suppressing NF-kappaB and NFATc1 activation and DC-STAMP expression. Acta Pharmacol Sin. (2016) 37:255–63. doi: 10.1038/aps.2015.85 PMC4753374 26592521

[26] Yu Y Wang D Li H Fan J Liu Y Zhao X. Mesenchymal stem cells derived from induced pluripotent stem cells play a key role in immunomodulation during cardiopulmonary resuscitation. Brain Res. (2019) 1720:146293. doi: 10.1016/j.brainres.2019.06.012 31201814

[27] Raschke WC Baird S Ralph P Nakoinz I. Functional macrophage cell lines transformed by Abelson leukemia virus. Cell. (1978) 15:261–7. doi: 10.1016/0092-8674(78)90101-0 212198

[28] Zheng H Li J Luo X Li C Hu L Qiu Q. Murine RAW264.7 cells as cellular drug delivery carriers for tumor therapy: a good idea? Cancer Chemother Pharmacol. (2019) 83:361–74. doi: 10.1007/s00280-018-3735-0 30506269

[29] Blasi E Mathieson BJ Varesio L Cleveland JL Borchert PA Rapp UR. Selective immortalization of murine macrophages from fresh bone marrow by a raf/myc recombinant murine retrovirus. Nature. (1985) 318:667–70. doi: 10.1038/318667a0 4079980

[30] Blasi E Barluzzi R Bocchini V Mazzolla R Bistoni F. Immortalization of murine microglial cells by a v-raf/v-myc carrying retrovirus. J Neuroimmunol. (1990) 27:229–37. doi: 10.1016/0165-5728(90)90073-V 2110186

[31] Cox GW Mathieson BJ Gandino L Blasi E Radzioch D Varesio L. Heterogeneity of hematopoietic cells immortalized by v-myc/v-raf recombinant retrovirus infection of bone marrow or fetal liver. J Natl Cancer Inst. (1989) 81:1492–6. doi: 10.1093/jnci/81.19.1492 2778838

[32] Roberson SM Walker WS. Immortalization of cloned mouse splenic macrophages with a retrovirus containing the v-raf/mil and v-myc oncogenes. Cell Immunol. (1988) 116:341–51. doi: 10.1016/0008-8749(88)90236-5 2460250

[33] Spera I Sánchez-Rodríguez R Favia M Menga A Venegas FC Angioni R. The J2-immortalized murine macrophage cell line displays phenotypical and metabolic features of primary BMDMs in their M1 and M2 polarization state. Cancers (Basel). (2021) 13:5478. doi: 10.3390/cancers13215478 PMC8582589 34771641

[34] Liu J Zhang Y Sheng H Liang C Liu H Moran Guerrero JA. Hyperoside suppresses renal inflammation by regulating macrophage polarization in mice with type 2 diabetes mellitus. Front Immunol. (2021) 12:733808. doi: 10.3389/fimmu.2021.733808 34925317 PMC8678409

[35] Maoldomhnaigh C Ó Cox DJ Phelan JJ Mitermite M Murphy DM Leisching G. Lactate alters metabolism in human macrophages and improves their ability to kill mycobacterium tuberculosis. Front Immunol. (2021) 12:663695. doi: 10.3389/fimmu.2021.663695 34691015 PMC8526932

[36] Rotondo R Barisione G Mastracci L Grossi F Orengo AM Costa R. IL-8 induces exocytosis of arginase 1 by neutrophil polymorphonuclears in nonsmall cell lung cancer. Int J Cancer. (2009) 125:887–93. doi: 10.1002/ijc.24448 19431148

[37] Joffe AM Bakalar MH Fletcher DA. Macrophage phagocytosis assay with reconstituted target particles. Nat Protoc. (2020) 15:2230–46. doi: 10.1038/s41596-020-0330-8 32561889

[38] Lamouille S Xu J Derynck R. Molecular mechanisms of epithelial-mesenchymal transition. Nat Rev Mol Cell Biol. (2014) 15:178–96. doi: 10.1038/nrm3758 PMC4240281 24556840

[39] Franklin RA Liao W Sarkar A Kim MV Bivona MR Liu K. The cellular and molecular origin of tumor-associated macrophages. Science. (2014) 344:921–5. doi: 10.1126/science.1252510 PMC4204732 24812208

[40] Zhao JL Ye YC Gao CC Wang L Ren KX Jiang R. Notch-mediated lactate metabolism regulates MDSC development through the Hes1/MCT2/c-Jun axis. Cell Rep. (2022) 38:110451. doi: 10.1016/j.celrep.2022.110451 35263597

[41] Locati M Mantovani A Sica A. Macrophage activation and polarization as an adaptive component of innate immunity. Adv Immunol. (2013) 120:163–84. doi: 10.1016/B978-0-12-417028-5.00006-5 24070384

[42] Xiang X Wang J Lu D Xu X. Targeting tumor-associated macrophages to synergize tumor immunotherapy. Signal Transduct Target Ther. (2021) 6:75. doi: 10.1038/s41392-021-00484-9 33619259 PMC7900181

[43] Mendoza R Banerjee I Manna D Reghupaty SC Yetirajam R Sarkar D. Mouse bone marrow cell isolation and macrophage differentiation. Methods Mol Biol. (2022) 2455:85–91. doi: 10.1007/978-1-0716-2128-8_8 35212988 PMC8936184

[44] Wang H Zhao C Santa-Maria CA Emens LA Popel AS. Dynamics of tumor-associated macrophages in a quantitative systems pharmacology model of immunotherapy in triple-negative breast cancer. iScience. (2022) 25:104702. doi: 10.1016/j.isci.2022.104702 35856032 PMC9287616

[45] Salem AM Mugundu GM Singh AP. Development of a multiscale mechanistic modeling framework integrating differential cellular kinetics of CAR T-cell subsets and immunophenotypes in cancer patients. CPT Pharmacometrics Syst Pharmacol. (2023) 12:1285–304. doi: 10.1002/psp4.13009 PMC10508581 37448297

